# Development of a multiplex RT-PCR assay for simultaneous detection of *Cucumber green mottle mosaic virus* and *Acidovorax citrulli* in watermelon

**DOI:** 10.7717/peerj.7539

**Published:** 2019-08-22

**Authors:** Xinyue Bi, Xiaodong Li, Haibo Yu, Mengnan An, Rui Li, Zihao Xia, Yuanhua Wu

**Affiliations:** 1 College of Plant Protection, Shenyang Agricultural University, Shenyang, Liaoning, China; 2 General Station of Forest and Grassland Pest and Diseases Control, National Forestry and Grassland Administration, Shenyang, Liaoning, China

**Keywords:** Multiplex RT-PCR, *Cucumber green mottle mosaic virus*, *Acidovorax citrulli*, Watermelon, Detection

## Abstract

Watermelon (*Citrullus lanatus* Thunb.) is considered as a popular and nutritious fruit crop worldwide. Watermelon blood flesh disease caused by *Cucumber green mottle mosaic virus* (CGMMV) and bacterial fruit blotch caused by *Acidovorax citrulli*, are two major quarantine diseases of watermelon and result in considerable losses to global watermelon production. In this study, a multiplex reverse-transcription polymerase chain reaction (RT-PCR) method was developed for simultaneous detection of CGMMV and *A. citrulli* in both watermelon leaves and seeds. Two pairs of specific primers were designed based on the conserved sequences of the genomic RNA of CGMMV and the internal transcribed spacer of *A. citrulli*, respectively. *Transcriptional elongation factor-1α* from watermelon was added as an internal reference gene to prevent false negatives. No cross-reactivity was detected with other viral or bacterial pathogens infecting watermelon. Moreover, the multiplex RT-PCR showed high sensitivity and could simultaneously detect CGMMV and *A. citrulli* as little as 10^2^ copies of plasmid DNA. This method was successfully applied to test field-collected watermelon leaves and stored seeds of cucurbitaceous crops. These results suggested that the developed multiplex RT-PCR technique is a rapid, efficient, and sensitive method for simultaneous detection of CGMMV and *A. citrulli*, providing technical support for monitoring, predicting, and preventing these two quarantine diseases. To our knowledge, this is the first report on simultaneous detection of a virus and a bacterium by multiplex RT-PCR in watermelon.

## Introduction

Watermelon (*Citrullus lanatus* Thunb.) is an economically important fruit crop worldwide (Guo et al., 2015). China has become the largest watermelon producing country, accounting for almost 67.1% of the total global watermelon production in 2017 (http://faostat.fao.org/). As with many other crops, watermelon production is threatened by pathogens, pests and abiotic stresses. Particularly, two plant diseases, blood flesh caused by *Cucumber green mottle mosaic virus* (CGMMV) and bacterial fruit blotch (BFB) caused by *Acidovorax citrulli*, have become predominant limiting factors in the yield and quality of watermelon in China (Li et al., 2017; Wu et al., 2016).

*Cucumber green mottle mosaic virus*, belonging to the genus *Tobamovirus*, is an important viral pathogen infecting cucurbitaceous crops, especially watermelon (Gibbs et al., 2015; Dombrovsky, Tran-Nguyen & Jones, 2017). Watermelon blood flesh caused by CGMMV is characterized by the purplish red or dark red coloration of the flesh adjacent to the seeds and leads to fruit decay with the typical mosaic patterning on the leaves of infected plants (Dombrovsky, Tran-Nguyen & Jones, 2017; Li et al., 2017). Recently, the rapid spread of CGMMV in watermelon has resulted in serious yield reduction and considerable economic losses, or even complete losses in severe regions such as Israel (Reingold et al., 2013) and Australia (Tesoriero et al., 2016). Additionally, a Gram-negative bacterium *A. citrulli* causes BFB and leads to serious losses to watermelon globally (Burdman & Walcott, 2012). *A. citrulli* usually causes irregularly shaped marks and water-soaked lesions on melon fruit, which can rapidly expand to cover the entire surface and lead to rot of fruit exocarp (Latin & Hopkins, 1995). Outbreaks of BFB can lead to 100% yield loss under suitable environmental conditions (Johnson et al., 2011). Considering the potential threat to the production of cucurbitaceous crops, CGMMV, and *A. citrulli* has been listed as plant quarantine pests in China (Hongyun et al., 2008; Tian et al., 2016).

Epidemiological characteristics of plant diseases caused by CGMMV and *A. citrulli* are involved in several common features. Both CGMMV and *A. citrulli* were seed-transmitted (Hollings, Komuro & Tochihara, 1975; Walcott & Gitaitis, 2000), caused huge damage and occurred in common disease regions; for example, North America, Asian, and Europe (Reingold et al., 2016; Dombrovsky, Tran-Nguyen & Jones, 2017; Tian et al., 2016). The infection rate of the secondary spread of CGMMV and *A. citrulli* was high and up to 86% and 95%, respectively (Reingold et al., 2016; Chalupowicz et al., 2015). Moreover, they could infect many of the same hosts, for example, watermelon, melon, and cucumber, etc. (Dombrovsky, Tran-Nguyen & Jones, 2017; Bahar, Kritzman & Burdman, 2009). Consequently, to establish a sensitive, reliable, and specific method is urgently required for quick and simultaneous detection of CGMMV and *A. citrulli* to prevent and monitor the spread of both pathogens.

Rapid and accurate pathogen detection is a crucial approach for disease diagnosis and management strategies to minimize damages caused by CGMMV and *A. citrulli* in watermelon and other cucurbitaceous crops. Recently, reverse-transcription polymerase chain reaction (RT-PCR)-based techniques have been developed to detect CGMMV alone, including RT-PCR assay (Li et al., 2018), immunocapture RT-PCR method (Shang et al., 2011) and real-time RT-PCR (Zhao et al., 2015). Currently, the molecular biological methods for detecting *A. citrulli* mainly included the conventional PCR (Bahar et al., 2008), real-time PCR (Tian et al., 2016), gold-labelled DNA strip sensor (Zhao et al., 2011), and cross-priming amplification (Zhang et al., 2012). However, no studies on simultaneous detection of CGMMV and *A. citrulli* in watermelon have been reported so far.

Specially, the requirement of amplification templates used in PCR method is different for detecting CGMMV and *A. citrulli*. For example, the detection of CGMMV regularly requires the extraction of RNA and subsequent cDNA synthesis by reverse transcription, while *A. citrulli* usually only needs the extracted DNA. To satisfy this different requirement of amplification templates, a multiplex RT-PCR for simultaneous detection of CGMMV and *A. citrulli* in watermelon was developed using reverse transcription products contained DNA and cDNA of total nucleic acids extracted from plant samples as templates. The total nucleic acid has been successfully used to detect strawberry RNA and DNA viruses (Chang et al., 2007), providing the feasibility of using total nucleic acid as a template for multiplex RT-PCR. Moreover, the reaction conditions of the multiplex RT-PCR were optimized, including concentration of primers, annealing temperature, extension time, and cycle number. The sensitivity and specificity of the primers used in the multiplex RT-PCR were assessed, respectively. Moreover, the developed multiplex RT-PCR was applied to test field-grown watermelon and other cucurbitaceous crop samples. To our knowledge, this is the first report on simultaneous detection of a virus and a bacterium in watermelon by multiplex RT-PCR. This study provides a rapid and feasible system for pathogen detection of CGMMV and *A. citrulli*, which contributed to plant disease diagnosis of watermelon blood flesh and BFB in watermelon as well as other cucurbitaceous crops.

## Materials and Methods

### Viruses and bacteria

The viruses CGMMV (CGMMV-lnxg; GenBank ID: KY040049.1), *Cucumber mosaic virus* (CMV) and the bacteria *A. citrulli* (JZ17; GenBank ID: MG655621), *Pseudomonas syringae* pv. *lachrymans* and *Xanthomonas campestris* pv. *cucurbitae* were stored in the Plant Virology laboratory at the College of Plant Protection, Shenyang Agricultural University. Other microorganisms, such as *Watermelon mosaic virus* (WMV), *Zucchini yellow mosaic virus* (ZYMV), and *Squash mosaic virus* (SqMV), were kindly provided by Dr. Qinsheng Gu (Zhengzhou Fruit Research Institute, CAAS).

### Plant material

Watermelon plants (*Citrullus lanatus* cv. Qilin) cultivated in greenhouse were mechanically inoculated with different kinds of viral and bacterial pathogens, and the samples were collected, immediately frozen in liquid nitrogen and stored at −80 °C until use. The leaves from field-grown watermelons used for disease diagnosis were collected from two crop fields: Xinmin city (42°03′22.21″N, 122°38′19.17″E) and Qingyuan city (42°05′10.70″N, 124°55′28.16″E), Liaoning Province, China. The seeds of cucurbitaceous crops used for pathogen detection were purchased randomly and the information was listed in Table S1.

### Design of primers

According to the genomic sequences of the CGMMV-lnxg isolate (GenBank ID: KY040049.1), the *A. citrulli* internal transcribed spacer (ITS; GenBank ID: MG655621), and the watermelon *transcriptional elongation factor-1α* (*EF-1α*; accession number: Cla010539, CuGenDB, http://www.cucurbitgenomics.org/), three pairs of specific primers, CGMMV-F/CGMMV-R, Ac-ITS-F/Ac-ITS-R, and SlEF-1α-F/SlEF-1α-R, were designed using Primer Premier 5.0 (Premier Biosoft International) and shown in Table 1. Their specificities were tested using NCBI Primer-BLAST (https://www.ncbi.nlm.nih.gov/tools/primer-blast/), respectively. The PCR amplification products of CGMMV and *A. citrulli* were 756 and 248 bp in size, respectively. In order to avoid false negative results, watermelon *EF-1α* was chosen as an internal control gene. Due to a 569-bp intron in DNA of *EF-1α*, the PCR amplification products of *EF-1α* were 540-bp by cDNA templates and 1,109-bp by DNA templates, which facilitates the visualization of multiple RT-PCR. All of primers were synthesized by Sangon Biotech Co. Ltd., Shanghai, China.

**Table 1:** Specific primers for multiplex RT-PCR.

Specific primer	Primer sequence (5′-3′)	Length (nt)	Size (bp)	GC (%)	Tm (°C)	Amplified gene
SlEF-1α-F	ACAGTCATTGATGCTCCCG	19	540, 1,109	52.6	55.1	Cla010539
SlEF-1α-R	GGTGACAACCATACCAGGCT	20		55.0	57.5	
Ac-ITS-F	CCTCCACCAACCAATACGCT	20	246	55.0	57.8	MG655621
Ac-ITS-R	GTCATTACTGAATTTCAACA	20		30.0	44.2	
CGMMV-F	TGGTGCTACTGTTGCTCTGG	20	756	55.0	57.6	KY040049.1
CGMMV-R	GAAGCGAAACGAAGATAGGC	20		50.0	53.7	

### Optimization of multiplex RT-PCR system

For total nucleic acid extraction, about 100 mg of samples were ground and homogenized in two mL of nucleic acid extraction solution A (each 100 mL of the extraction solution contained two g sodium dodecyl sulfate (SDS), 1.5 g PVP-40, 0.9 g NaCl, 6.2 g NaAc · 3H_2_O, five mL HAc, and four mL of 0.5 mol L^−1^ Methylenediaminetetraacetic acid; the solution was incubated at 35 °C in a water bath for 15 min and shaken well before use), followed by 0.1 g sample and one g quartz sand. The samples were ground into a homogeneous mixture, and 500 μL of the mixture was transferred into a 1.5 mL centrifuge tube and incubated in a water bath at 65 °C for 30 min. Next, 200 μL nucleic acid extraction solution B (each 100 mL solution contained 29.4 g KAc and 11.5 mL HAc) was added, and the solution was mixed and incubated at −20 °C for 5 min. Subsequently, the solution was shaken by inverting, and 750 μL chloroform was added. The solution was shaken and centrifuged at 4 °C at 12,000 rpm for 20 min. Next, 500 μL supernatant was obtained, and 400 μL chloroform and 400 μL water-saturated phenol were added. The solution was shaken well and then centrifuged at 4 °C at 12,000 rpm for 15 min. The supernatant was collected, and an equal volume of isopropanol was added. The solution was well mixed and then incubated at −20 °C for 20 min. Subsequently, the solution was centrifuged at 4 °C at 12,000 rpm for 15 min. The supernatant was discarded, and the pellet was rinsed using one mL anhydrous ethanol. After the ethanol was removed, the pellet was dried at room temperature, and the nucleic acid was dissolved in 20 μL diethyl pyrocarbonate-treated water. Using a micro-volume spectrophotometer (Thermo Fisher Scientific, Waltham, MA, USA), the A260/A280 values of total DNA and RNA were 1.99–2.03 and 2.01–2.05, respectively; and the A260/A230 values of total DNA and RNA were 1.79–1.94 and 1.79–1.92, respectively. Two microliters of total nucleic acid (approximately 1.5 μg RNA) was collected as the template, to which 0.5 μL of Oligo dT (0.5 μg μL^−1^; Sangon Biotech, Shanghai, China), 0.5 μL random primers (0.5 μg μL^−1^; Sangon Biotech, Shanghai, China), and one μL dNTPs (10 mmol L^−1^; Sangon Biotech, Shanghai, China) were added. DEPC-water was added until the total volume reached 14.5 μL. The solution was incubated at 70 °C for 5 min and then placed on ice for 3–5 min. Four microliters of 5× reverse transcription buffer (Sangon Biotech, Shanghai, China), 0.5 μL ribonuclease inhibitor (40 U μL^−1^; Sangon Biotech, Shanghai, China), and one μL M-MLV reverse transcriptase (200 U μL^−1^; Sangon Biotech, Shanghai, China) were added. The solution was mixed well and then placed in a water bath at 42 °C for 1 h. The obtained cDNA was stored at –20 °C.

The total volume of multiplex RT-PCR reaction was 25 μL, including one μL of template (reverse transcription products of total nucleic acids), upstream and downstream primers (Table 2), 12.5 μL of 2× TaKaRa Premix (Ex Taq, 1.25 U/25 μL; dNTP Mixture, each 0.4 mmol L^−1^; Mg^2+^, 4 mmol L^−1^), and appropriate ddH_2_O. The PCR procedures were as follows: pre-denaturation at 94 °C for 5 min; 15, 20, 25, 30, 35, or 45 cycles of denaturation at 94 °C for 30 s, annealing at varying temperatures (48.2, 48.9, 50.1, 51.6, 53.6, 55.9, 58.0, 59.6, 60.9, or 61.6 °C) for 30 s, and extension at 72 °C for various durations (15, 25, 35, 45, 60, or 80 s); and a final extension at 72 °C for 10 min.

**Table 2:** Final concentrations of primers.

Test number	Primer concentration (μmol μL^−1^)		
	CGMMV-F/CGMMV-R	Ac-ITS-F/Ac-ITS-R	SlEF-1α-F/SlEF-1α-R
1	0.2	0.2	0.2
2	0.4	0.2	0.2
3	0.2	0.1	0.2
4	0.1	0.1	0.2
5	0.2	0.2	0.4
6	0.2	0.1	0.4
7	0.1	0.1	0.4

The amplification products of multiplex RT-PCR were obtained using a TIANgel Midi Purification Kit (Tiangen Biotech, Beijing, China). Purified PCR products were cloned and constructed pEASY-T1-CGMMV, pEASY-T1-*A. citrulli*, and pEASY-T1-*EF-1α* (540-bp) plasmid by using a pEASY-T1 Simple Cloning Kit (TransGen Biotech, Beijing, China), respectively. Positive clones were further verified using colony PCR and the plasmids were submitted for sequencing (Sangon Biotech, Shanghai, China).

### Specificity and sensitivity of the multiplex RT-PCR

Watermelon leaves infected by various viruses, including CMV, WMV, ZYMV, and SqMV, and by two bacteria, including *P. syringae* pv. *lachrymans*, and *X. campestris* pv. *cucurbitae*, were examined to verify the specificity of the multiplex RT-PCR system. Amplified reaction was conducted using the optimized multiplex PCR system and conditions. Furthermore, a series of 10-fold dilutions from 1 × 10^10^ to 1 × 10^0^ copies was performed by using the templates (mixture of pEASY-T1-CGMMV, pEASY-T1-*A. citrulli* and pEASY-T1-*EF-1α* plasmids, with the concentration of 50.6 ng μL^−1^, 48.4 ng μL^−1^ and 45.1 ng μL^−1^, respectively) to determine the lowest detection limit. PCR products were separated by 1.5% agarose gel electrophoresis, and a gel imaging system (Bio-Rad, Hercules, CA, USA) was employed to observe the electrophoresis results.

### Enzyme-linked immunosorbent detection

Dot enzyme-linked immunosorbent assay (Dot-ELISA) and double antibody sandwich enzyme-linked immunosorbent assay (Das-ELISA) were performed to detect CGMMV and *A. citrulli* in these seed samples of cucurbitaceous crops, respectively. The seeds were ground in the TBS buffer (20 mM Tris-HCl, 0.15 M NaCl, pH 8.0) by a ratio of 1:6 (w/v). For Dot-ELISA, monoclonal antibody of CGMMV was used as previously reported (Shang et al., 2011). For Das-ELISA, the detection kit (Agdia, Elkhart, IN, USA) for *A. citrulli* was used.

## Results

### Specificity of multiplex RT-PCR

**Figure 1:**
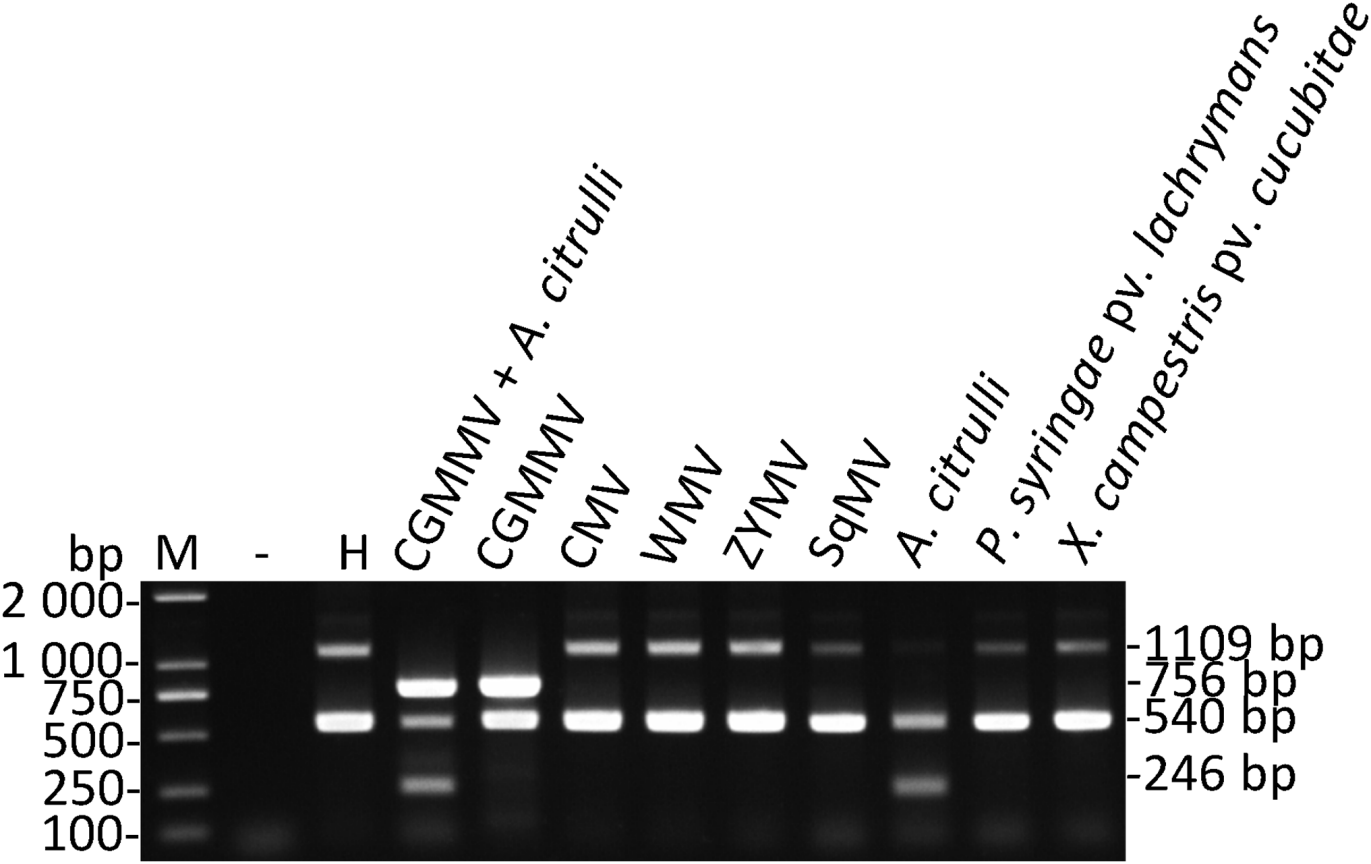
Specificity of multiplex RT-RCR. The watermelon leaves infected by CMV, WMV, ZYMV, SqMV, *P. syringae* pv. *lachrymans* and *X. campestris* pv. *cucubitae* were used to assess the specificity of the primers used for multiplex RT-PCR, respectively. Lane −, ddH_2_O; lane H, healthy watermelon leaves; lane M, *Trans* 2K DNA marker. CGMMV, *Cucumber green mottle mosaic virus*; CMV, *Cucumber mosaic virus*; WMV, *Watermelon mosaic virus*; ZYMV, *Zucchini yellow mosaic virus*; SqMV, *Squash mosaic virus*; *A. citrulli*, *Acidovorax citrulli*; *P. syringae* pv. *Lachrymans*, *Pseudomonas syringae* pv. *lachrymans*; *X. campestris* pv. *cucubitae*, *Xanthomonas campestris* pv. *cucurbitae*.

Total nucleic acids extracted from the watermelon leaves infected by CMV, WMV, ZYMV, SqMV, *P. syringae* pv. *lachrymans*, and *X. campestris* pv. *cucurbitae* were performed to assess the specificity of the primers used for multiplex RT-PCR (Fig. 1). Three amplified products (756, 246, and 540 bp) were detected in the sample mixture of watermelon leaves infected by CGMMV or *A. citrulli*. Moreover, the corresponding amplification segment of 756 or 246 bp in length was showed in watermelon samples infected by CGMMV or *A. citrulli*, while the segments of watermelon *EF-1α* were observed only in the watermelon leaves infected by other pathogens (e.g., CMV, WMV, ZYMV, SqMV, *P. syringae* pv. *lachrymans*, and *X. campestris* pv. *cucurbitae*) or healthy watermelon leaves (Fig. 1). Unexpectedly, the 1,109-bp segment of *EF-1α* was weak or undetectable in the presence of CGMMV or *A. citrulli* or both, which had no effects on the results of pathogen detection. These results demonstrated that the developed multiplex RT-PCR system was highly specific to simultaneously detect CGMMV and *A. citrulli*.

The amplification products of CGMMV, *A. citrulli* ITS and watermelon *EF-1α* (540 bp) generated by multiplex RT-PCR were cloned and sequenced. The results showed that the obtained fragments had over 99% nucleotide sequence identity (CGMMV, identity 99.63%; *A. citrulli* ITS, identity 100%; watermelon *EF-1α*, identity 100%) with the known sequences, suggesting that the primers were highly specific and the detection results of multiplex RT-PCR were reliable.

### Optimization of reaction condition in multiplex RT-PCR

**Figure 2:**
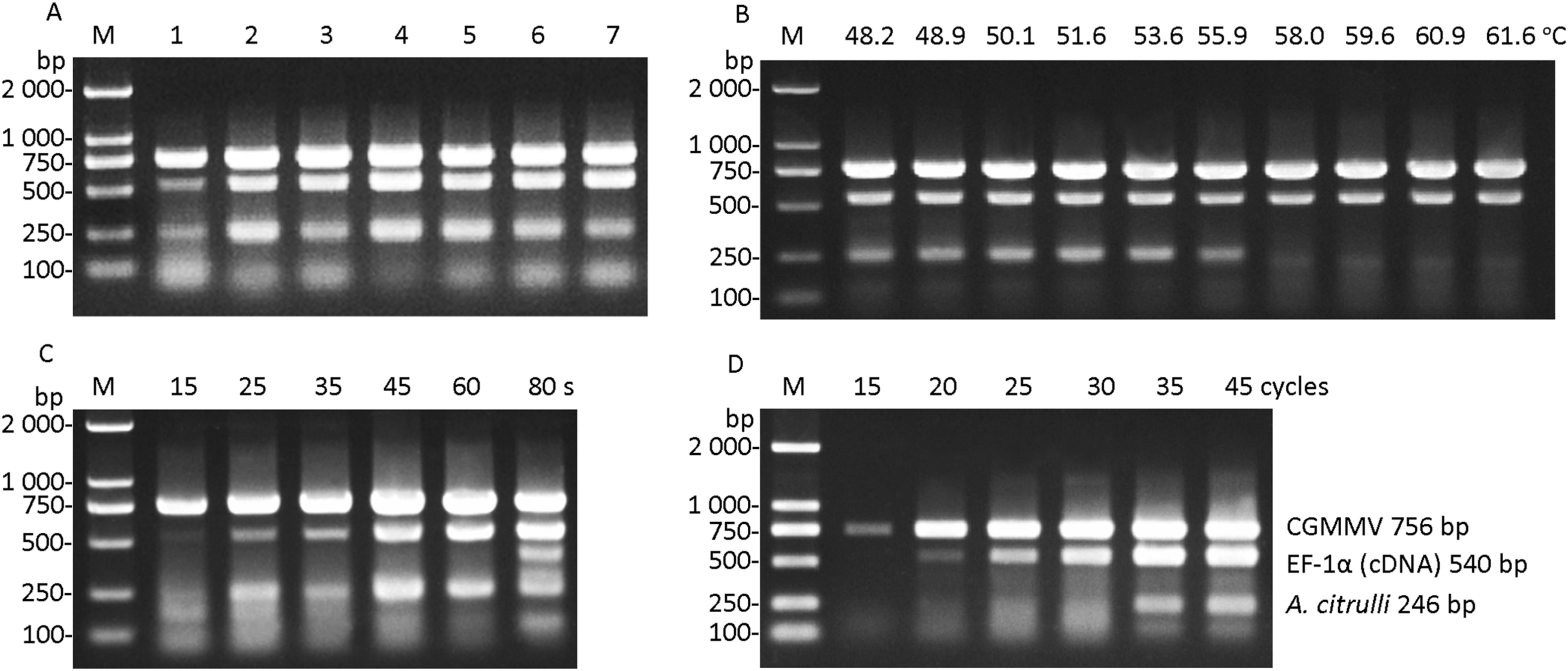
Optimization of reaction conditions in multiplex RT-PCR. (A) Optimization of concentrations of primers. Lanes 1-7, test number 1-7 shown in Table 2, respectively. (B) Optimization of annealing temperature from 48.2 to 61.6 °C. (C) Optimization of extension time from 15 to 80 s. (D) Optimization of cycle number from 15 to 45 cycles. Lane M, *Trans* 2K DNA marker. CGMMV, *Cucumber green mottle mosaic virus*; *A. citrulli*, *Acidovorax citrulli*; *EF-1α*, *transcriptional elongation factor-1α*.

The optimization of the primer concentrations showed in Table 2 was performed. The results showed that high amplification efficiency and low primer dimers were observed in line 4 (Fig. 2A), of which the final concentrations of primers for CGMMV, *A. citrulli* ITS and watermelon *EF-1α* were determined as 0.1, 0.1, and 0.2 μmol μL^−1^, respectively. The results of annealing temperature optimization demonstrated that the amplification product of *A. citrulli* was weak and unclear when the annealing temperature was higher than 55.9 °C (Fig. 2B). To ensure the amplification efficiency, the annealing temperature at 54 °C was finally selected for the multiplex RT-PCR. In the assay of extension time optimization, 45 or 60 s as extension time permitted effective detection of the target segments of CGMMV, *A. citrulli* ITS, and watermelon *EF-1α* (Fig. 2C). In consideration of time saving, 45 s was chosen as the extension time. For cycle number optimization, effective detection of target segments of CGMMV, *A. citrulli* ITS, and watermelon *EF-1α* was observed when the cycle number was 35 or 40 (Fig. 2D). Thus, to make the assay time-saving and more efficient, we chose 35 cycles for the multiplex RT-PCR. In conclusion, the optimized multiplex RT-PCR was carried out with appropriate primer concentration, annealing temperature at 54 °C, extension time for 45 s with 35 cycles.

### Sensitivity of multiplex RT-PCR

**Figure 3:**
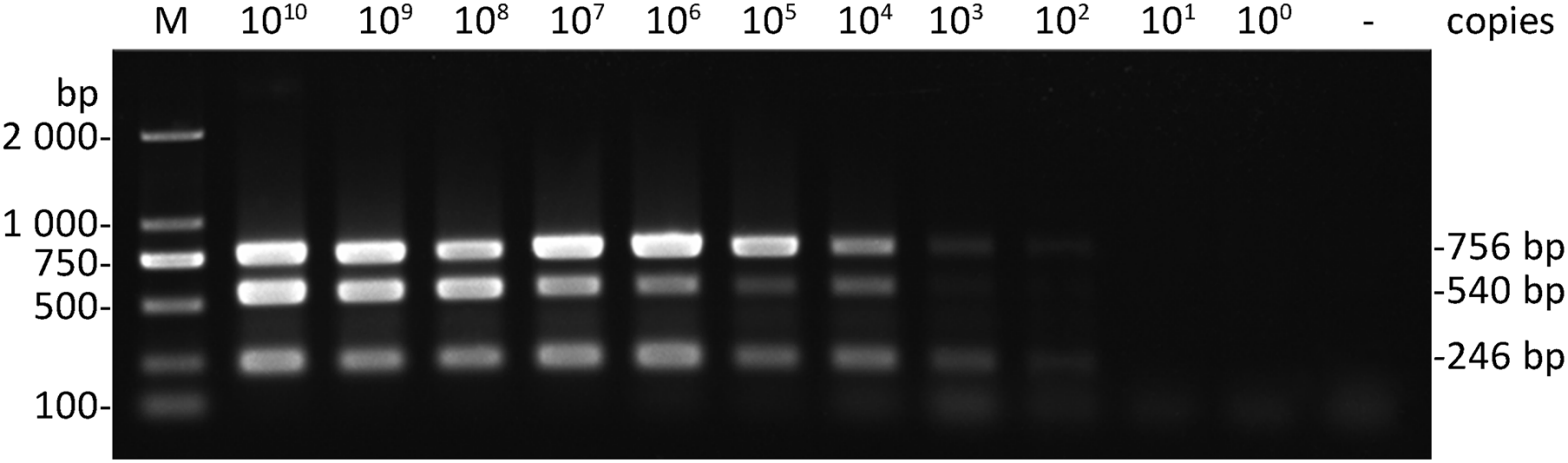
Sensitivity of multiplex RT-RCR. Correspond to serial 10-fold dilutions of the plasmids (pEASY-T1-CGMMV, 50.6 ng μL^−1^; pEASY-T1-*A. citrulli*, 48.4 ng μL^−1^; pEASY-T1-*EF-1α*, 45.1 ng μL^−1^) ranging from 1 × 10^10^ to 1 × 10^0^ copies. Lane −, ddH_2_O; lane M, *Trans* 2K DNA marker.

To determine the sensitivity of multiplex RT-PCR, a series of 10-fold dilutions of the mixture contained three kinds of plasmids (pEASY-T1-CGMMV, pEASY-T1-*A. citrulli* and pEASY-T1-*EF-1α*) were tested by the optimized system of multiplex RT-PCR. The results showed that the developed multiplex RT-PCR method could simultaneously detect CGMMV and *A. citrulli* as little as both 10^2^ copies (0.51 and 0.48 fg uL^−1^, respectively) of plasmid DNA, and *EF-1α* up to 10^3^ copies (4.51 fg uL^−1^) (Fig. 3), indicating that the developed multiplex RT-PCR method in this study was highly sensitive for simultaneous detection of CGMMV and *A. citrulli*.

### Disease diagnosis of field-grown samples and pathogen detection of seeds by multiplex RT-PCR

**Figure 4:**
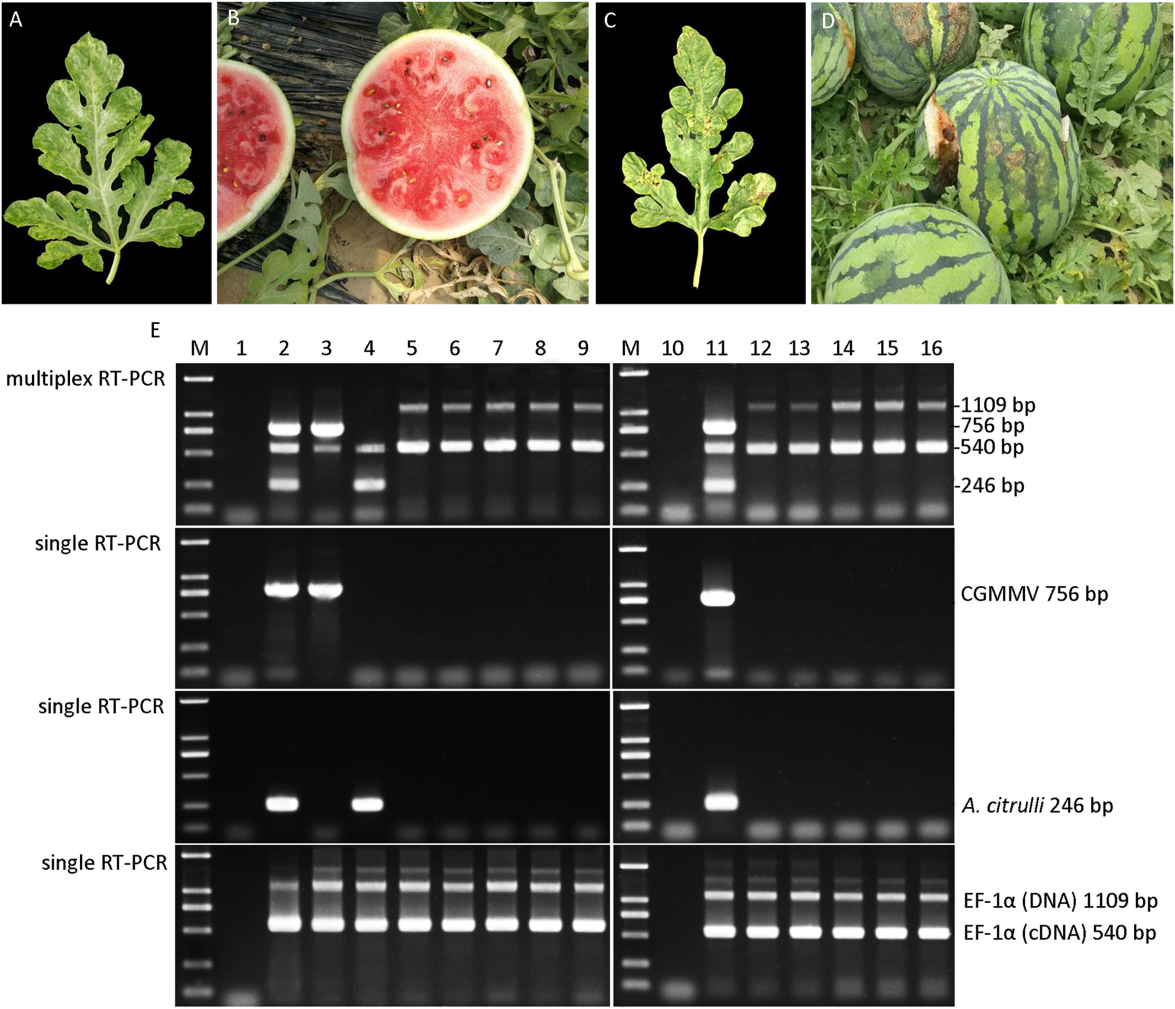
Disease diagnosis of field-grown watermelon samples by multiplex RT-PCR. (A) Leaf sample suspected with CGMMV infection. (B) Fruit sample suspected with CGMMV infection. (C) Leaf sample suspected with *A. citrulli* infection. (D) Fruit sample suspected with *A. citrulli* infection. (E) Diagnosis of field-grown watermelon samples using multiplex RT-PCR and single RT-PCR. Lanes 1 and 10, ddH_2_O; lanes 2 and 11, mixture of CGMMV- and *A. citrulli*-infected watermelon leaves; lane 3, suspected CGMMV-infected leaf sample; lane 4, suspected *A. citrulli*-infected leaf sample; lanes 5–9, random leaf samples; lanes 12–16, random seed samples; Lane M, *Trans* 2K DNA marker.

To evaluate the feasibility of the established multiplex RT-PCR for simultaneous detection of CGMMV and *A. citrulli* in the field, multiplex and single RT-PCR were performed using the watermelon samples with the symptoms of CGMMV or *A. citrulli*-like (Figs. 4A–4D). The results of multiplex RT-PCR assessment revealed that CGMMV was detected in the watermelon leaves from Xinmin city, whilst *A. citrulli* was identified in the samples collected from Qingyuan city (Fig. 4E). However, neither CGMMV nor *A. citrulli* was found in other five watermelon leaves and seeds sampled randomly. Additionally, the data of single RT-PCR provided strong support for the results of multiplex RT-PCR (Fig. 4E). These results demonstrated that the multiplex RT-PCR method was successfully applied for the detection of these two quarantine pathogens in the field, which contributed to the diagnosis and monitoring epidemic pattern of these two watermelon diseases.

**Figure 5:**
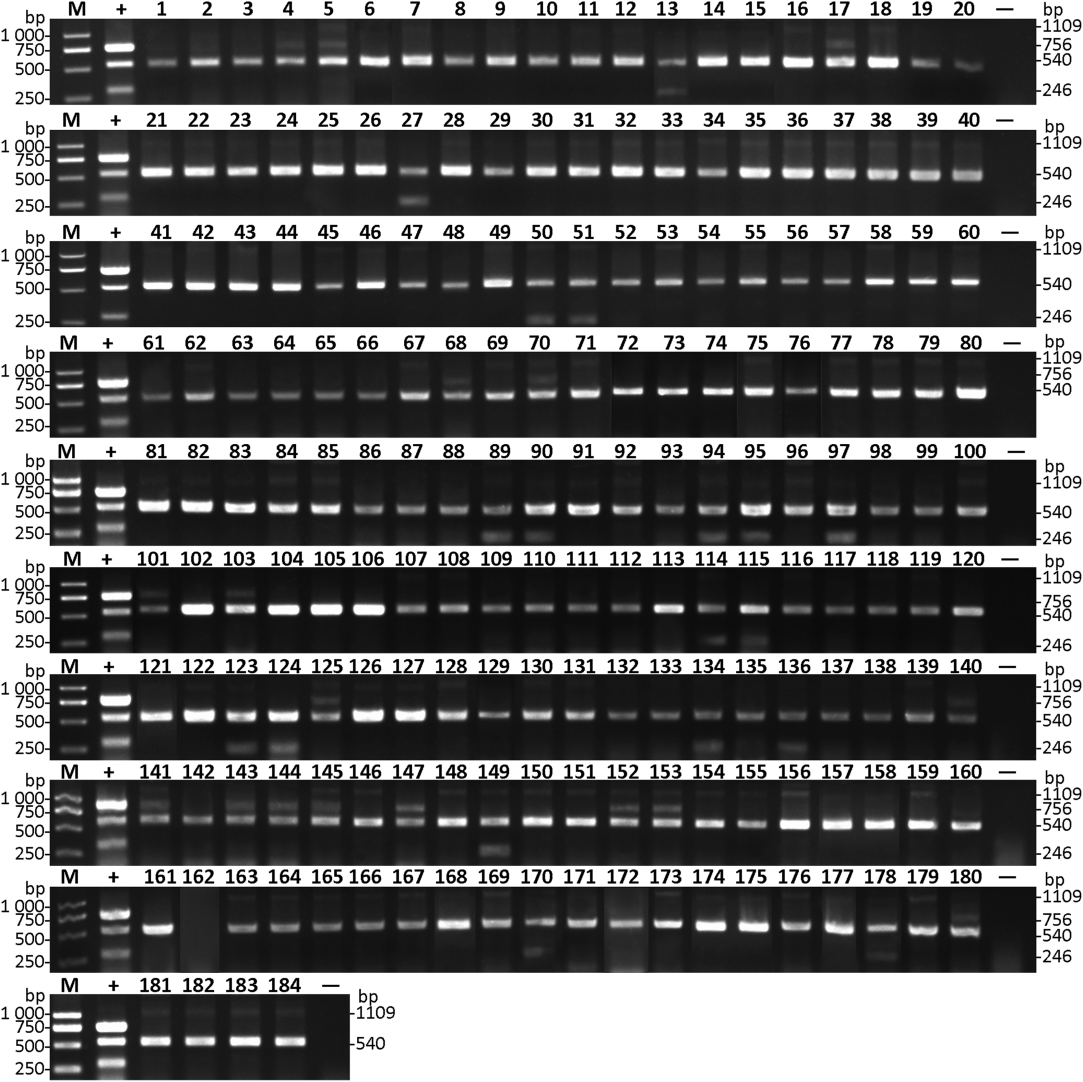
Detection of CGMMV and *A. citrulli* in seeds of cucurbitaceous crops using multiplex RT-PCR. Lanes 1–184, seed samples of cucurbitaceous crops listed in Table S1; lane +, mixture of CGMMV- and *A. citrulli*-infected watermelon leaves; lane −, ddH_2_O; lane M, *Trans* 2K DNA marker.

Considering the seed transmission of both CGMMV and *A. citrulli*, 184 varieties of seeds of cucurbitaceous crops from Liaoning province were collected and tested by multiplex RT-PCR (Table S1). The results showed that CGMMV was detected in 17 varieties of seeds (Fig. 5), which were mainly from *Cucurbita moschata* (Duch. ex Lam.) and *Citrullus lanatus* (Thunb.), and distributed in Xinmin city (Table S1). Meanwhile, 18 varieties of seeds carrying *A. citrulli* were detected (Fig. 5), including *Citrullus lanatus* (Thunb.) and *Cucumis melo* Linn., which were mainly distributed in Shuncheng and Qingyuan city (Table S1). Both CGMMV and *A. citrulli* were detected in the regions of Shuncheng, Jiangping, Tai’an and Xinmin city (Table S1). Moreover, the detection results of multiplex RT-PCR were verified by Dot-ELISA for CGMMV (Fig. S1) and Das-ELISA for *A. citrulli* (Table S2). The results also revealed that multiplex RT-PCR was more sensitive than Dot-ELISA and similar with Das-ELISA (Table S1). These results demonstrated that the established multiplex RT-PCR can be used for high-throughput screening for seeds of cucurbitaceous crops.

## Discussion

CGMMV and *A. citrulli* are two major quarantine pathogens infecting watermelon, which cause extensive damage and huge economic losses to farmers, and the diseases caused by these two pathogens have broken out recently (Dombrovsky, Tran-Nguyen & Jones, 2017; Silva et al., 2016). Both of two pathogens are seed-transmitted (Hollings, Komuro & Tochihara, 1975; Walcott & Gitaitis, 2000) and have many of the same hosts (Dombrovsky, Tran-Nguyen & Jones, 2017; Bahar, Kritzman & Burdman, 2009). Therefore, it is significant to develop a multiplex RT-PCR system to simultaneously diagnose watermelon blood flesh and BFB in field-grown samples, and detect CGMMV and *A. citrulli* in seeds of watermelon and other cucurbitaceous crops for monitoring, predicting, and preventing these two quarantine diseases.

In this study, a multiplex RT-PCR method was established to simultaneously detect CGMMV and *A. citrulli*, which was successfully applied to field-grown samples and stored seeds. The detection methods of CGMMV or *A. citrulli* were widely reported, including one-step reverse transcription loop-mediated isothermal amplification for CGMMV detection (Li et al., 2013), multiplex RT-PCR assays for simultaneous detection of seven cucurbit-infecting viruses including CGMMV (Kwon et al., 2014), an ELISA-based assay for detection of *A. citrulli* (Himananto et al., 2011), a microsphere immunoassay (MIA) for simultaneous detection of multiple plant pathogens, including *A. citrulli* (Charlermroj et al., 2017). However, the detection object of these reported methods cannot give consideration to both CGMMV and *A. citrulli*, especially PCR-based methods, probable due to the difficulty of acquirement of suitable amplification template for a RNA virus and a bacterium. Although several methods have been reported for detecting both plant bacteria and viruses simultaneously, they are all less-than-perfect. Recently, a method based on a MIA has been developed for multiplex detection of three viruses (potyviruses, *Watermelon silver mottle virus*, *Melon yellow spot virus*) and *A. citrulli* in cucurbits (Charlermroj et al., 2017). However, it required the specific antibodies that were expensive. Especially, MIA was not as sensitive as RT-PCR. The multiplex PCR method was developed for simultaneous detection of four viruses and a bacterium in citrus (Meena & Baranwal, 2016). The total RNA and DNA was extracted separately from different infected plants, and the acquired cDNA and DNA was mixed and used as templates in multiplex PCR reaction mixture. In our study, only the total nucleic acid contained DNA and RNA was used as the template for multiplex RT-PCR, which was more convenient and faster. To our best knowledge, this is the first report on simultaneous detection of a virus and a bacterium in watermelon by multiplex RT-PCR method.

In our study, the total nucleic acids contained DNA and RNA were extracted from watermelon leaves and seeds using a modified SDS method with high quality, safety, and economy. The RNase, polysaccharide and proteins existed in the leaves and seeds of watermelon could be effectively eliminated by utilizing the SDS and HAc-NaAC buffer system. Moreover, it was simple that the total nucleic acids were extracted by one-step method and the reverse transcription products were directly used as the templates for multiplex RT-PCR. Unfortunately, it was difficult to accurately measure the initial concentration of DNA and RNA of the total nucleic acid extracted by this method. Therefore, the sensitivity of the multiplex RT-PCR was quantitatively verified by the copy number of plasmids (Fig. 3). However, this did not affect the utilization of the developed multiplex RT-PCR method for simultaneous detection of CGMMV and *A. citrulli*.

Primer is a key factor for establishing an efficient multiplex RT-PCR method. Primer design and selection need be in consideration of specificity and sensitivity of the primers, potential secondary structure formation between primer sets, and distinguishable amplicon sizes. In this study, the primers CGMMV-F/R were designed according to the conserved regions of CGMMV genome with a low mutation rate, which were located in the regions of movement protein (MP) and coat protein (CP) gene (from 5,260 to 6,015 nt). The primers can detect CGMMV with high sensitivity and produce a 756-bp fragment, which ensured the specificity of primers and visualization of the multiplex RT-PCR results (Fig. 1). To detect CGMMV, several primers have been reported in different detection methods, including CGMMV-primers based on MP and CP gene (from 4,989 to 6,258 nt) for conventional RT-PCR that produced a 1,269-bp band (Tesoriero et al., 2016), and CGMMV-CP-RT-primers based on CP gene for real-time-qPCR method generating a 141-bp fragment (Lee et al., 2016). With comparing the ITS regions of several bacteria in the genus *Acidovorax*, primers Ac-ITS-F/R designed based on the 16S-23S ITS region of the ribosomal DNA sequence of *A. citrulli* according to the previous report (Walcott, Gitaitis & Castro, 2003), which can distinguish *A. citrulli* among different bacteria of the genus *Acidovorax*, such as *A. avenae*, *A. cattelyeae*, *A. konjaci*, *A. facilis*, etc. (Walcott, Gitaitis & Castro, 2003), and produced products with the expected sizes of 246 bp (Fig. 1). ITS region of 16S-23S rDNA sequences has been reported to be used for specific primer design to detect *A. citrulli* in watermelon seeds sensitively and specifically (Schaad, Song & Hatziloukas, 2000). A set of LAMP primers FIP/BIP based on the *hrpG-hrpX* gene spacer region were also designed for specific detection of *A. citrulli* on seeds of cucurbitaceous crops (Oya et al., 2008). Primers SlEF-1α-F/R were designed to amplify watermelon *EF-1α* gene, which could efficiently prevent false negatives. *EF-1α* gene was also used as the internal control in multiplex RT-PCR for detection of four apple viruses (Ji et al., 2013). In this work, due to the existence of a 569-bp intron, the PCR amplification products of *EF-1α* were two fragments that were 540-bp by cDNA template and 1,109-bp by DNA template (Table 1). However, the 1,109-bp segment was weak or undetectable in the presence of pathogens (Fig. 1), possibly because the DNA templates were diluted during reverse transcription. Moreover, the presence of CGMMV and *A. citrulli* resulted in competition for PCR resources, which affected the amplification efficiency of the watermelon *EF-1α* 1,109-bp fragment. Notably, this did not affect the detection of these two pathogens.

Additionally, success of the multiplex RT-PCR assay also depends on the sensitivity of methods. In this study, the developed multiplex RT-PCR method could simultaneously detect CGMMV and *A. citrulli* as little as 10^2^ copies of plasmid DNA (Fig. 3), which was similar to that of the RT-LAMP detection method for CMV (Peng et al., 2012). Moreover, in the CGMMV detection assays of cucurbitaceous seeds, 17 of 184 varieties showed positive results by multiplex RT-PCR (Fig. 5), of which only 10 samples were positive by Dot-ELISA (Table S1), suggesting that the multiplex RT-PCR showed higher sensitivity than Dot-ELISA. These results indicated that the sensitivity of the established multiplex RT-PCR method was sufficient to detect these two pathogens in the cucurbitaceous crops, which could efficiently prevent and monitor the spread of both diseases.

In view of the seed-transmitted feature of CGMMV and *A. citrulli* (Hollings, Komuro & Tochihara, 1975; Walcott & Gitaitis, 2000), the multiplex RT-PCR method in our study can be widely used for seed quarantine, which had the prominent advantages of high efficiency, low cost, time saving and high sensitivity. In this study, we successfully tested a large number of seed samples from different regions by multiplex RT-PCR (Fig. 5), providing technical support for monitoring, predicting, and preventing the potential risk of these two quarantine diseases. Therefore, this method is helpful for field disease management, selection of disease-resistant varieties, and restriction of disease spread.

## Conclusions

In summary, a multiplex RT-PCR assay was developed for rapid and sensitive detection of CGMMV and *A. citrulli* in field-grown watermelon samples and stored cucurbitaceous seeds to prevent the possible spread of these two pathogens. To our knowledge, this is the first report on simultaneous detection of a virus and a bacterium in watermelon by multiplex RT-PCR method.

## Supplemental Information

10.7717/peerj.7539/supp-1 Supplemental Information 1 Detection of CGMMV and *A. citrulli* in seed samples of cucurbitaceous crops using ELISA and multiplex RT-PCR.

10.7717/peerj.7539/supp-2 Supplemental Information 2 Detection of *Acidovorax citrulli* in seeds of cucurbitaceous crops using Das-ELISA.

10.7717/peerj.7539/supp-3 Supplemental Information 3 Detection of CGMMV in seeds of cucurbitaceous crops using Dot-ELISA.

10.7717/peerj.7539/supp-4 Supplemental Information 4 Original Gel Figure of Detection of CGMMV and *A. citrulli* in seeds of cucurbitaceous crops using multiplex RT-PCR.

10.7717/peerj.7539/supp-5 Supplemental Information 5 The updated lanes in Fig. 5.

## Additional Information and Declarations

### Data Availability

The following information was supplied regarding data availability: The raw measurements are available in the Supplemental Files.
