# Supplementary material for: Development of a multiplex RT-PCR assay for simultaneous detection of Cucumber green mottle mosaic virus and Acidovorax citrulli in watermelon
Source: PeerJ. 2019 Aug 22;7:e7539. doi: 10.7717/peerj.7539 (PMC6708580; doi:10.7717/peerj.7539)
Supplement: Supplemental Information 1 [file peerj-07-7539-s001.docx]

Table S1 Detection of CGMMV and *A. citrulli* in seed samples of cucurbitaceous crops using ELISA and multiplex RT-PCR.

| Sample | Code name | Crop species | Sampling spot | CGMMV | | *A. citrulli* | |
| --- | --- | --- | --- | --- | --- | --- | --- |
|  |  |  |  | RT-PCR | Dot-ELISA | RT-PCR | Das-ELISA |
| 1 | BZ-1 | *Cucurbita moschata* (Duch. ex Lam.) | Beizhen | − | − | − | − |
| 2 | BZ-2 | *Cucurbita moschata* (Duch. ex Lam.) | (41°35′32.96″N, 121°47′35.30″E) | − | − | − | − |
| 3 | BZ-3 | *Cucurbita moschata* (Duch. ex Lam.) |  | − | − | − | − |
| 4 | BZ-4 | *Cucurbita moschata* (Duch. ex Lam.) |  | + | + | − | − |
| 5 | BZ-5 | *Citrullus lanatus* (Thunb.) |  | + | − | − | − |
| 6 | FC-1 | *Citrullus lanatus* (Thunb.) | Fengcheng | − | − | − | − |
| 7 | FC-2 | *Cucumis sativus* Linn. | (40°27′4.48″N, 124°03′36.85″E) | − | − | − | − |
| 8 | FC-3 | *Cucumis sativus* Linn. |  | − | − | − | − |
| 9 | FC-4 | *Cucumis sativus* Linn. |  | − | − | − | − |
| 10 | FC-5 | *Cucumis sativus* Linn. |  | − | − | − | − |
| 11 | FC-6 | *Cucumis sativus* Linn. |  | − | − | − | − |
| 12 | FC-7 | *Cucumis sativus* Linn. |  | − | − | − | − |
| 13 | KZ-1 | *Cucumis melo* Linn | Kazuo | − | − | + | + |
| 14 | KZ-2 | *Citrullus lanatus* (Thunb.) | (41°35′50.59″N, 120°24′35.19″E) | − | − | − | − |
| 15 | KZ-3 | *Citrullus lanatus* (Thunb.) |  | − | − | − | − |
| 16 | JC-1 | *Cucumis sativus* Linn. | Jianchang | − | − | − | − |
| 17 | JC-2 | *Cucumis sativus* Linn. | (40°49′21.20″N, 119°49′52.80″E) | + | + | − |  |
| 18 | JC-3 | *Cucumis sativus* Linn. |  | − | − | − | − |
| 19 | JC-4 | *Cucumis sativus* Linn. |  | − | − | − | − |
| 20 | JC-5 | *Cucumis sativus* Linn. |  | − | − | − | − |
| 21 | JC-6 | *Cucumis sativus* Linn. |  | − | − | − | − |
| 22 | JC-7 | *Cucumis sativus* Linn. |  | − | − | − | − |
| 23 | JC-8 | *Cucumis sativus* Linn. |  | − | − | − | − |
| 24 | JC-9 | *Cucumis sativus* Linn. |  | − | − | − | − |
| 25 | PLD-1 | *Cucumis sativus* Linn. | Pulandian | − | − | − | − |
| 26 | PLD-2 | *Cucumis sativus* Linn. | (39°23′36.44″N,121°57′29.41″E） | − | − | − | − |
| 27 | PLD-3 | *Cucumis melo* Linn. |  | − | − | + | + |
| 28 | PLD-4 | *Cucumis sativus* Linn. |  | − | − | − | − |
| 29 | PLD-5 | *Cucumis sativus* Linn. |  | − | − | − | − |
| 30 | PLD-6 | *Cucumis sativus* Linn. |  | − | − | − | − |
| 31 | PLD-7 | *Cucumis sativus* Linn. |  | − | − | − | − |
| 32 | PLD-8 | *Cucumis sativus* Linn. |  | − | − | − | − |
| 33 | PLD-9 | *Cucumis sativus* Linn. |  | − | − | − | − |
| 34 | PLD-10 | *Citrullus lanatus* (Thunb.) |  | − | − | − | − |
| 35 | PLD-11 | *Cucumis sativus* Linn. |  | − | − | − | − |
| 36 | PLD-12 | *Cucumis sativus* Linn. |  | − | − | − | − |
| 37 | PLD-13 | *Cucumis sativus* Linn. |  | − | − | − | − |
| 38 | PLD-14 | *Cucumis sativus* Linn. |  | − | − | − | − |
| 39 | PLD-15 | *Cucumis sativus* Linn. |  | − | − | − | − |
| 40 | PLD-16 | *Cucumis sativus* Linn. |  | − | − | − | − |
| 41 | PLD-17 | *Cucumis sativus* Linn. |  | − | − | − | − |
| 42 | PLD-18 | *Cucumis sativus* Linn. |  | − | − | − | − |
| 43 | PLD-19 | *Cucumis sativus* Linn. |  | − | − | − | − |
| 44 | PLD-20 | *Cucumis sativus* Linn. |  | − | − | − | − |
| 45 | PLD-21 | *Citrullus lanatus* (Thunb.) |  | − | − | − | − |
| 46 | PLD-22 | *Cucumis sativus* Linn. |  | − | − | − | − |
| 47 | SZ-1 | *Citrullus lanatus* (Thunb.) | Suizhong | − | − | − | − |
| 48 | SZ-2 | *Cucumis melo* Linn. | (40°23′3.26″N, 120°08′39.64″E) | − | − | − | − |
| 49 | SZ-3 | Cucurbita pepo Linn. |  | − | − | − | − |
| 50 | SZ-4 | *Citrullus lanatus* (Thunb.) |  | − | − | + | + |
| 51 | SZ-5 | *Citrullus lanatus* (Thunb.) |  | − | − | + | + |
| 52 | ZW-1 | *Cucurbita moschata* (Duch. ex Lam.) | Zhangwu | − | − | − | − |
| 53 | ZW-2 | *Citrullus lanatus* (Thunb.) | (42°22′36.59″N, 122°28′57.56″E) | − | − | − | − |
| 54 | ZW-3 | *Cucurbita moschata* (Duch. ex Lam.) |  | − | − | − | − |
| 55 | ZW-4 | *Citrullus lanatus* (Thunb.) |  | − | − | − | − |
| 56 | ZW-5 | *Cucurbita moschata* (Duch. ex Lam.) |  | − | − | − | − |
| 57 | ZW-6 | *Cucurbita moschata* (Duch. ex Lam.) |  | − | − | − | − |
| 58 | FC-1 | *Citrullus lanatus* (Thunb.) | Fumeng | − | − | − | − |
| 59 | FC-2 | *Cucurbita moschata* (Duch. ex Lam.) | (42°04′43.96″N, 121°45′9.58″E) | − | − | − | − |
| 60 | FC-3 | *Citrullus lanatus* (Thunb.) |  | − | − | − | − |
| 61 | FC-4 | *Cucurbita moschata* (Duch. ex Lam.) |  | − | − | − | − |
| 62 | XF-1 | *Citrullus lanatus* (Thunb.) | Xifeng | − | − | − | − |
| 63 | XF-2 | *Cucurbita moschata* (Duch. ex Lam.) | (42°44′36.30″N, 124°43′19.87″E) | − | − | − | − |
| 64 | XF-3 | *Cucurbita moschata* (Duch. ex Lam.) |  | − | − | − | − |
| 65 | XF-4 | *Cucurbita moschata* (Duch. ex Lam.) |  | − | − | − | − |
| 66 | HR-1 | *Cucurbita moschata* (Duch. ex Lam.) | Hengren | − | − | − | − |
| 67 | HR-2 | *Citrullus lanatus* (Thunb.) | (41°16′47.87″N, 125°21′5.32″E) | − | − | − | − |
| 68 | ST-1 | *Cucurbita moschata* (Duch. ex Lam.) | Shuangta | + | + | − | − |
| 69 | ST-2 | *Citrullus lanatus* (Thunb.) | (41°32′31.81″N, 120°29′14.11″E) | − | − | − | − |
| 70 | LS-1 | *Citrullus lanatus* (Thunb.) | Lianshan | + | − | − | − |
| 71 | LS-2 | *Citrullus lanatus* (Thunb.) | (40°46′25.17″N, 120°51′51.35″E) | − | − | − | − |
| 72 | DSQ-1 | *Lagenaria siceraria* (Molina) | Dashiqiao | − | − | − | − |
| 73 | DSQ-2 | *Lagenaria siceraria* (Molina) | (40°40′30.64″N, 122°29′12.02″E) | − | − | − | − |
| 74 | DSQ-3 | *Lagenaria siceraria* (Molina) |  | − | − | − | − |
| 75 | DSQ-4 | *Citrullus lanatus* (Thunb.) |  | − | − | − | − |
| 76 | DSQ-5 | *Citrullus lanatus* (Thunb.) |  | − | − | − | − |
| 77 | DSQ-6 | *Cucurbita moschata* (Duch. ex Lam.) |  | − | − | − | − |
| 78 | DSQ-7 | *Citrullus lanatus* (Thunb.) |  | − | − | − | − |
| 79 | BX-1 | *Citrullus lanatus* (Thunb.) | Benxi | − | − | − | − |
| 80 | BX-2 | *Cucumis sativus* Linn. | (41°18′58.56″N, 124°05′19.27″E) | − | − | − | − |
| 81 | BX-3 | *Cucumis sativus* Linn. |  | − | − | − | − |
| 82 | BX-4 | *Cucumis sativus* Linn. |  | − | − | − | − |
| 83 | FS-1 | *Cucumis melo* Linn. | Fushun | − | − | − | − |
| 84 | FS-2 | *Citrullus lanatus* (Thunb.) | (41°47′15.84″N, 124°11′2.01″E) | − | − | − | − |
| 85 | FS-3 | *Citrullus lanatus* (Thunb.) |  | − | − | − | − |
| 86 | SC-1 | *Cucurbita moschata* (Duch. ex Lam.) | Shuncheng | − | − | − | − |
| 87 | SC-2 | *Cucurbita moschata* (Duch. ex Lam.) | (41°54′8.04″N, 123°55′21.80″E) | − | − | − | − |
| 88 | SC-3 | *Cucurbita moschata* (Duch. ex Lam.) |  | − | − | − | − |
| 89 | SC-4 | *Citrullus lanatus* (Thunb.) |  | − | − | + | + |
| 90 | SC-5 | *Cucumis melo* Linn. |  | − | − | + | + |
| 91 | SC-6 | *Cucumis melo* Linn. |  | − | − | − | − |
| 92 | SC-7 | *Citrullus lanatus* (Thunb.) |  | − | − | − | − |
| 93 | SC-8 | *Cucurbita moschata* (Duch. ex Lam.) |  | − | − | − | − |
| 94 | SC-9 | *Citrullus lanatus* (Thunb.) |  | − | − | + | + |
| 95 | SC-10 | *Cucumis melo* Linn. |  | − | − | + | − |
| 96 | SC-11 | *Citrullus lanatus* (Thunb.) |  | − | − | − | − |
| 97 | SC-12 | *Cucumis melo* Linn. |  | − | − | + | − |
| 98 | SC-13 | *Cucurbita moschata* (Duch. ex Lam.) |  | − | − | − | − |
| 99 | SC-14 | *Cucurbita moschata* (Duch. ex Lam.) |  | − | − | − | − |
| 100 | SC-15 | *Citrullus lanatus* (Thunb.) |  | − | − | − | − |
| 101 | SC-16 | *Cucurbita moschata* (Duch. ex Lam.) |  | + | − | − | − |
| 102 | SC-17 | *Cucumis melo* Linn. |  | − | − | − | − |
| 103 | SC-18 | *Cucumis melo* Linn. |  | + | + | − | − |
| 104 | XB-1 | *Cucumis melo* Linn. | Xinbin | − | − | − | − |
| 105 | XB-2 | *Cucumis melo* Linn. | (41°43′52.76″N, 125°02′1.25″E) | − | − | − | − |
| 106 | XB-3 | *Cucumis melo* Linn. |  | − | − | − | − |
| 107 | XB-4 | *Citrullus lanatus* (Thunb.) |  | − | − | − | − |
| 108 | QY-1 | *Citrullus lanatus* (Thunb.) | Qingyuan | − | − | − | − |
| 109 | QY-2 | *Cucumis melo* Linn. | (42°05′9.72″N, 124°55′27.26″E) | − | − | − | − |
| 110 | QY-3 | *Cucurbita moschata* (Duch. ex Lam.) |  | − | − | − | − |
| 111 | QY-4 | *Cucurbita moschata* (Duch. ex Lam.) |  | − | − | − | − |
| 112 | QY-5 | *Cucurbita moschata* (Duch. ex Lam.) |  | − | − | − | − |
| 113 | QY-6 | *Cucumis melo* Linn. |  | − | − | − | − |
| 114 | QY-7 | *Citrullus lanatus* (Thunb.) |  | − | − | + | + |
| 115 | QY-8 | *Cucumis melo* Linn. |  | − | − | + | + |
| 116 | QY-9 | *Citrullus lanatus* (Thunb.) |  | − | − | − | + |
| 117 | QY-10 | *Cucurbita moschata* (Duch. ex Lam.) |  | − | − | − | − |
| 118 | WFD-1 | *Citrullus lanatus* (Thunb.) | Wafangdian | − | − | − | − |
| 119 | WFD-2 | *Citrullus lanatus* (Thunb.) | (39°37′54.29″N, 121°57′39.86″E) | − | − | − | − |
| 120 | WFD-3 | *Lagenaria siceraria* (Molina) |  | − | − | − | − |
| 121 | WFD-4 | *Lagenaria siceraria* (Molina) |  | − | − | − | − |
| 122 | JP-1 | *Cucumis sativus* Linn. | Jianping | − | − | − | − |
| 123 | JP-2 | *Cucumis sativus* Linn. | (41°24′35.69″N, 119°37′37.29″E) | − | − | + | + |
| 124 | JP-3 | *Cucumis melo* Linn. |  | − | − | + | + |
| 125 | JP-4 | *Cucurbita pepo* Linn. |  | + | + | − | − |
| 126 | JP-5 | *Cucurbita moschata* (Duch. ex Lam.) |  | − | − | − | − |
| 127 | CY-1 | *Cucumis sativus* Linn. | Chaoyang | − | − | − | − |
| 128 | CY-2 | *Citrullus lanatus* (Thunb.) | (41°22′18.71″N, 120°17′10.09″E) | − | − | − | − |
| 129 | DW-1 | *Cucurbita moschata* (Duch. ex Lam.) | Dawaxian | − | − | − | − |
|  |  |  | (41°0′1.75″N, 122°04′37.14″E) |  |  |  |  |
| 130 | Y-1 | *Citrullus lanatus* (Thunb.) | Yi | − | − | − | − |
|  |  |  | (41°31′51.34″N, 121°14′1.04″E) |  |  |  |  |
| 131 | XM-1 | *Citrullus lanatus* (Thunb.) | Xinmin | − | − | − | − |
| 132 | XM-2 | *Citrullus lanatus* (Thunb.) | (42°0′32.44″N, 122°47′51.02″E) | − | − | − | − |
| 133 | XM-3 | *Citrullus lanatus* (Thunb.) |  | − | − | − | − |
| 134 | XM-4 | *Cucurbita moschata* (Duch. ex Lam.) |  | − | − | + | + |
| 135 | XM-5 | *Citrullus lanatus* (Thunb.) |  | − | − | − | − |
| 136 | XM-6 | *Citrullus lanatus* (Thunb.) |  | − | − | + | + |
| 137 | XM-7 | *Citrullus lanatus* (Thunb.) |  | − | − | − | − |
| 138 | XM-8 | *Citrullus lanatus* (Thunb.) |  | − | − | − | − |
| 139 | XM-9 | *Citrullus lanatus* (Thunb.) |  | − | − | − | − |
| 140 | XM-10 | *Cucurbita moschata* (Duch. ex Lam.) |  | + | + | − | − |
| 141 | XM-11 | *Citrullus lanatus* (Thunb.) |  | + | − | − | − |
| 142 | XM-12 | *Cucurbita moschata* (Duch. ex Lam.) |  | − | − | − | − |
| 143 | XM-13 | *Cucurbita moschata* (Duch. ex Lam.) |  | + | + | − | − |
| 144 | XM-14 | *Cucurbita moschata* (Duch. ex Lam.) |  | + | + | − | − |
| 145 | XM-15 | *Citrullus lanatus* (Thunb.) |  | + | + | − | − |
| 146 | XM-16 | *Citrullus lanatus* (Thunb.) |  | − | − | − | − |
| 147 | XM-17 | *Cucurbita moschata* (Duch. ex Lam.) |  | + | − | − | − |
| 148 | XM-18 | *Citrullus lanatus* (Thunb.) |  | − | − | − | − |
| 149 | XM-19 | *Citrullus lanatus* (Thunb.) |  | − | − | + | − |
| 150 | XM-20 | *Citrullus lanatus* (Thunb.) |  | − | − | − | − |
| 151 | XM-21 | *Citrullus lanatus* (Thunb.) |  | − | − | − | − |
| 152 | XM-22 | *Citrullus lanatus* (Thunb.) |  | + | − | − | − |
| 153 | XM-23 | *Citrullus lanatus* (Thunb.) |  | + | − | − | − |
| 154 | XM-24 | *Citrullus lanatus* (Thunb.) |  | − | − | − | − |
| 155 | HC-1 | *Citrullus lanatus* (Thunb.) | Haicheng | − | − | − | − |
| 156 | HC-2 | *Citrullus lanatus* (Thunb.) | (40°53′52.07″N, 122°39′11.57″E) | − | − | − | − |
| 157 | HC-3 | *Lagenaria siceraria* (Molina) |  | − | − | − | − |
| 158 | HC-4 | *Cucurbita moschata* (Duch. ex Lam.) |  | − | − | − | − |
| 159 | HC-5 | *Citrullus lanatus* (Thunb.) |  | − | − | − | − |
| 160 | BP-1 | *Cucurbita moschata* (Duch. ex Lam.) | Beipiao | − | − | − | − |
| 161 | BP-2 | *Lagenaria siceraria* (Molina) | (41°49′7.81″N, 120°44′51.97″E) | − | − | − | − |
| 162 | BP-3 | *Momordica charantia* Linn. |  | − | − | − | − |
| 163 | CT-1 | *Citrullus lanatus* (Thunb.) | Changtu | − | − | − | − |
| 164 | CT-2 | *Citrullus lanatus* (Thunb.) | (42°47′49.03″N, 124°05′13.63″E) | − | − | − | − |
| 165 | CT-3 | *Citrullus lanatus* (Thunb.) |  | − | − | − | − |
| 166 | PJ-1 | *Citrullus lanatus* (Thunb.) | Panjin | − | − | − | − |
| 167 | PJ-2 | *Citrullus lanatus* (Thunb.) | (41°14′31.76″N, 121°53′39.70″E) | − | − | − | − |
| 168 | PJ-3 | *Lagenaria siceraria* (Molina) |  | − | − | − | − |
| 169 | PJ-4 | *Citrullus lanatus* (Thunb.) | Xingcheng | − | − | − | − |
| 170 | PJ-5 | *Cucumis melo* Linn. | (40°36′35.18″N, 120°41′23.28″E) | − | − | + | + |
| 171 | PJ-6 | *Cucurbita moschata* (Duch. ex Lam.) |  | − | − | − | − |
| 172 | GZ-1 | *Lagenaria siceraria* (Molina) | Gaizhou | − | − | − | − |
| 173 | GZ-2 | *Citrullus lanatus* (Thunb.) | (40°23′5.52″N, 122°17′20.98″E) | − | − | − | − |
| 174 | GZ-3 | *Lagenaria siceraria* (Molina) |  | − | − | − | − |
| 175 | GZ-4 | *Lagenaria siceraria* (Molina) |  | − | − | − | − |
| 176 | GZ-5 | *Citrullus lanatus* (Thunb.) |  | − | − | − | − |
| 177 | TA-1 | *Lagenaria siceraria* (Molina) | Taian | − | − | − | − |
| 178 | TA-2 | *Citrullus lanatus* (Thunb.) | (41°24′51.82″N, 122°25′23.35″E) | − | − | + | + |
| 179 | TA-3 | *Citrullus lanatus* (Thunb.) |  | − | − | − | − |
| 180 | TA-4 | *Cucurbita moschata* (Duch. ex Lam.) |  | + | + | − | − |
| 181 | JZ-1  1 | *Citrullus lanatus* (Thunb.) | Jinzhou | − | − | − | − |
| 182 | JZ-2 | *Citrullus lanatus* (Thunb.) | (41°16′41.80″N, 120°54′36.01″E) | − | − | − | − |
| 183 | JZ-3 | *Citrullus lanatus* (Thunb.) |  | − | − | − | − |
| 184 | JZ-4 | *Citrullus lanatus* (Thunb.) |  | − | − | − | − |

+, positive result (■, RT-PCR; ■, Dot-ELISA; ■, Das-ELISA); −, negative result.
