# Supplementary material for: Development of a multiplex RT-PCR assay for simultaneous detection of Cucumber green mottle mosaic virus and Acidovorax citrulli in watermelon
Source: PeerJ. 2019 Aug 22;7:e7539. doi: 10.7717/peerj.7539 (PMC6708580; doi:10.7717/peerj.7539)
Supplement: Supplemental Information 2 [file peerj-07-7539-s002.docx]

Table S2 Detection of *Acidovorax citrulli* in seeds of cucurbitaceous crops using Das-ELISA

| Seed sample number | | | | | | | | | Reading values of ELISA | | | | | | | | |
| --- | --- | --- | --- | --- | --- | --- | --- | --- | --- | --- | --- | --- | --- | --- | --- | --- | --- |
| 1 | 1 | 1 | 2 | 2 | 2 | 3 | 3 | 3 | 0.1212 | 0.1159 | 0.1211 | 0.1086 | 0.1084 | 0.1066 | 0.1048 | 0.1099 | 0.1006 |
| 4 | 4 | 4 | 5 | 5 | 5 | 6 | 6 | 6 | 0.1057 | 0.1038 | 0.1086 | 0.1038 | 0.1037 | 0.1027 | 0.1007 | 0.1025 | 0.1016 |
| 7 | 7 | 7 | 8 | 8 | 8 | 9 | 9 | 9 | 0.1019 | 0.1048 | 0.1005 | 0.0987 | 0.0958 | 0.1011 | 0.1002 | 0.0988 | 0.1003 |
| 10 | 10 | 10 | 11 | 11 | 11 | 12 | 12 | 12 | 0.1015 | 0.1066 | 0.1013 | 0.0959 | 0.0997 | 0.0996 | 0.0988 | 0.1004 | 0.1086 |
| 13 | 13 | 13 | 14 | 14 | 14 | 15 | 15 | 15 | 0.3759^+^ | 0.3792^+^ | 0.3542^+^ | 0.0982 | 0.1004 | 0.1002 | 0.0999 | 0.0999 | 0.1006 |
| 16 | 16 | 16 | 17 | 17 | 17 | 18 | 18 | 18 | 0.0996 | 0.0995 | 0.1052 | 0.1074 | 0.0911 | 0.1124 | 0.0779 | 0.0975 | 0.1487 |
| 19 | 19 | 19 | 20 | 20 | 20 | 21 | 21 | 21 | 0.0782 | 0.0862 | 0.0767 | 0.1044 | 0.1042 | 0.1049 | 0.1068 | 0.1054 | 0.0956 |
| 22 | 22 | 22 | 23 | 23 | 23 | 24 | 24 | 24 | 0.0868 | 0.0905 | 0.0796 | 0.0878 | 0.0995 | 0.0756 | 0.0846 | 0.0956 | 0.8546 |
| 25 | 25 | 25 | 26 | 26 | 26 | 27 | 27 | 27 | 0.1132 | 0.1037 | 0.1132 | 0.1079 | 0.1102 | 0.1086 | 0.2044^+^ | 0.2102^+^ | 0.2313^+^ |
| 28 | 28 | 28 | 29 | 29 | 29 | 30 | 30 | 30 | 0.0973 | 0.1068 | 0.1086 | 0.1113 | 0.1144 | 0.1134 | 0.1208 | 0.1077 | 0.1065 |
| 31 | 31 | 31 | 32 | 32 | 32 | 33 | 33 | 33 | 0.1152 | 0.1137 | 0.1131 | 0.1089 | 0.1112 | 0.1096 | 0.1077 | 0.1083 | 0.1077 |
| 34 | 34 | 34 | 35 | 35 | 35 | 36 | 36 | 36 | 0.1059 | 0.1112 | 0.1085 | 0.1068 | 0.1069 | 0.1036 | 0.1155 | 0.1146 | 0.1133 |
| 37 | 37 | 37 | 38 | 38 | 38 | 39 | 39 | 39 | 0.1337 | 0.1380 | 0.1355 | 0.1079 | 0.1075 | 0.1057 | 0.1047 | 0.1072 | 0.1074 |
| 40 | 40 | 40 | 41 | 41 | 41 | 42 | 42 | 42 | 0.1091 | 0.1138 | 0.1071 | 0.1195 | 0.1195 | 0.1180 | 0.1303 | 0.1138 | 0.4341 |
| 43 | 43 | 43 | 44 | 44 | 44 | 45 | 45 | 45 | 0.1133 | 0.1137 | 0.1157 | 0.1088 | 0.1055 | 0.1097 | 0.1042 | 0.1067 | 0.1109 |
| 46 | 46 | 46 | 47 | 47 | 47 | 48 | 48 | 48 | 0.1091 | 0.1116 | 0.0991 | 0.1099 | 0.0984 | 0.0893 | 0.0826 | 0.0947 | 0.1207 |
| 49 | 49 | 49 | 50 | 50 | 50 | 51 | 51 | 51 | 0.1081 | 0.1016 | 0.0981 | 0.5109^+^ | 0.7618^+^ | 0.9270^+^ | 0.2548^+^ | 0.2026^+^ | 0.2933^+^ |
| 52 | 52 | 52 | 53 | 53 | 53 | 54 | 54 | 54 | 0.1012 | 0.0722 | 0.0704 | 0.0722 | 0.0701 | 0.0877 | 0.0723 | 0.0711 | 0.0855 |
| 55 | 55 | 55 | 56 | 56 | 56 | 57 | 57 | 57 | 0.0974 | 0.1152 | 0.0716 | 0.1089 | 0.1087 | 0.1053 | 0.0943 | 0.0939 | 0.0742 |
| 58 | 58 | 58 | 59 | 59 | 59 | 60 | 60 | 60 | 0.1135 | 0.1181 | 0.1148 | 0.1067 | 0.1043 | 0.1065 | 0.1219 | 0.1132 | 0.1154 |
| 61 | 61 | 61 | 62 | 62 | 62 | 63 | 63 | 63 | 0.1205 | 0.1194 | 0.1217 | 0.1112 | 0.1172 | 0.1089 | 0.1203 | 0.0977 | 0.0802 |
| 64 | 64 | 64 | 65 | 65 | 65 | 66 | 66 | 66 | 0.1033 | 0.1151 | 0.0846 | 0.0817 | 0.0811 | 0.0904 | 0.0886 | 0.1128 | 0.0901 |
| 67 | 67 | 67 | 68 | 68 | 68 | 69 | 69 | 69 | 0.0977 | 0.0911 | 0.0842 | 0.0956 | 0.0847 | 0.0953 | 0.0968 | 0.0933 | 0.0845 |
| 70 | 70 | 70 | 71 | 71 | 71 | 72 | 72 | 72 | 0.0843 | 0.1005 | 0.1087 | 0.0739 | 0.0770 | 0.0789 | 0.0778 | 0.0767 | 0.0755 |
| 73 | 73 | 73 | 74 | 74 | 74 | 75 | 75 | 75 | 0.0737 | 0.0752 | 0.0740 | 0.1145 | 0.1214 | 0.1088 | 0.0753 | 0.0826 | 0.0734 |
| 76 | 76 | 76 | 77 | 77 | 77 | 78 | 78 | 78 | 0.1105 | 0.0981 | 0.1197 | 0.1092 | 0.1092 | 0.1089 | 0.1098 | 0.1107 | 0.1105 |
| 79 | 79 | 79 | 80 | 80 | 80 | 81 | 81 | 81 | 0.1114 | 0.0922 | 0.1167 | 0.1098 | 0.1105 | 0.1133 | 0.1114 | 0.1086 | 0.1114 |
| 82 | 82 | 82 | 83 | 83 | 83 | 84 | 84 | 84 | 0.0984 | 0.1116 | 0.1136 | 0.1134 | 0.1107 | 0.1078 | 0.1101 | 0.1104 | 0.0984 |
| 85 | 85 | 85 | 86 | 86 | 86 | 87 | 87 | 87 | 0.1106 | 0.1242 | 0.1145 | 0.1180 | 0.1162 | 0.1144 | 0.1088 | 0.1138 | 0.1106 |
| 88 | 88 | 88 | 89 | 89 | 89 | 90 | 90 | 90 | 0.0786 | 0.0749 | 0.0810 | 0.4303^+^ | 0.4138^+^ | 0.4341^+^ | 0.4230^+^ | 0.4136^+^ | 0.4085^+^ |
| 91 | 91 | 91 | 92 | 92 | 92 | 93 | 93 | 93 | 0.0778 | 0.0733 | 0.0759 | 0.1110 | 0.1111 | 0.1105 | 0.1181 | 0.1153 | 0.1121 |
| 94 | 94 | 94 | 95 | 95 | 95 | 96 | 96 | 96 | 0.9061^+^ | 0.8238^+^ | 0.9855^+^ | 0.1100 | 0.1198 | 0.1175 | 0.1109 | 0.1109 | 0.1139 |
| 97 | 07 | 97 | 98 | 98 | 98 | 99 | 99 | 99 | 0.1311 | 0.1266 | 0.1259 | 0.1101 | 0.1096 | 0.1097 | 0.1096 | 0.1108 | 0.1110 |
| 100 | 100 | 100 | 101 | 101 | 101 | 102 | 102 | 102 | 0.1051 | 0.1051 | 0.1049 | 0.1079 | 0.1073 | 0.1104 | 0.1099 | 0.1087 | 0.1089 |
| 103 | 103 | 103 | 104 | 104 | 104 | 105 | 105 | 105 | 0.1275 | 0.1152 | 0.1069 | 0.1085 | 0.1082 | 0.1128 | 0.1086 | 0.1072 | 0.1090 |
| 106 | 106 | 106 | 107 | 107 | 107 | 108 | 108 | 108 | 0.1051 | 0.1023 | 0.1111 | 0.1109 | 0.1165 | 0.1074 | 0.1296 | 0.1229 | 0.1151 |
| 109 | 109 | 109 | 110 | 110 | 110 | 111 | 111 | 111 | 0.1026 | 0.1017 | 0.1016 | 0.1103 | 0.1199 | 0.1192 | 0.1135 | 0.1187 | 0.1137 |
| 112 | 112 | 112 | 113 | 113 | 113 | 114 | 114 | 114 | 0.1024 | 0.1017 | 0.1038 | 0.1005 | 0.1080 | 0.1103 | 0.4102^+^ | 0.9907^+^ | 0.8343^+^ |
| 115 | 115 | 115 | 116 | 116 | 116 | 117 | 117 | 117 | 0.1893^+^ | 0.2226^+^ | 0.3347^+^ | 0.2043^+^ | 0.1939^+^ | 0.1742^+^ | 0.1334 | 0.1323 | 0.1113 |
| 118 | 118 | 118 | 119 | 119 | 119 | 120 | 120 | 120 | 0.1084 | 0.1039 | 0.1093 | 0.1114 | 0.1163 | 0.1125 | 0.1135 | 0.1278 | 0.1084 |
| 121 | 121 | 121 | 122 | 122 | 122 | 123 | 123 | 123 | 0.1086 | 0.1117 | 0.1094 | 0.1098 | 0.1205 | 0.1076 | 0.4384^+^ | 0.3959^+^ | 0.4248^+^ |
| 124 | 134 | 124 | 125 | 125 | 125 | 126 | 126 | 126 | 0.2558^+^ | 0.5416^+^ | 0.4176^+^ | 0.1141 | 0.1077 | 0.1100 | 0.1169 | 0.1218 | 0.1225 |
| 127 | 127 | 127 | 128 | 128 | 128 | 129 | 129 | 129 | 0.1039 | 0.1209 | 0.1166 | 0.1115 | 0.1123 | 0.1103 | 0.1018 | 0.1156 | 0.1039 |
| 130 | 130 | 130 | 131 | 131 | 131 | 132 | 132 | 132 | 0.1093 | 0.1104 | 0.1256 | 0.1130 | 0.1111 | 0.1112 | 0.1041 | 0.1090 | 0.1093 |
| 133 | 133 | 133 | 134 | 134 | 134 | 135 | 135 | 135 | 0.0793 | 0.0956 | 0.1197 | 0.4843^+^ | 0.5505^+^ | 0.3087^+^ | 0.0825 | 0.1211 | 0.1024 |
| 136 | 136 | 136 | 137 | 137 | 137 | 138 | 138 | 138 | 0.2445^+^ | 0.2514^+^ | 0.2588^+^ | 0.1064 | 0.0995 | 0.1048 | 0.1014 | 0.1098 | 0.1123 |
| 139 | 139 | 139 | 140 | 140 | 140 | 141 | 141 | 141 | 0.0836 | 0.1013 | 0.1079 | 0.1220 | 0.1121 | 0.1027 | 0.0975 | 0.1254 | 0.1136 |
| 142 | 142 | 142 | 143 | 143 | 143 | 144 | 144 | 144 | 0.1061 | 0.1075 | 0.1084 | 0.1108 | 0.1065 | 0.1113 | 0.1060 | 0.1113 | 0.1061 |
| 145 | 145 | 145 | 146 | 146 | 146 | 147 | 147 | 147 | 0.1055 | 0.1019 | 0.0978 | 0.1021 | 0.1031 | 0.1049 | 0.1174 | 0.1161 | 0.1055 |
| 148 | 148 | 148 | 149 | 149 | 149 | 150 | 150 | 150 | 0.1075 | 0.1038 | 0.1028 | 0.1101 | 0.1077 | 0.1102 | 0.1011 | 0.1015 | 0.1075 |
| 151 | 151 | 151 | 152 | 152 | 152 | 153 | 153 | 153 | 0.098 | 0.0972 | 0.1041 | 0.0966 | 0.1208 | 0.1015 | 0.1101 | 0.1189 | 0.098 |
| 154 | 154 | 154 | 155 | 155 | 155 | 156 | 156 | 156 | 0.1005 | 0.0984 | 0.098 | 0.1 | 0.1027 | 0.1 | 0.0994 | 0.0981 | 0.1005 |
| 157 | 157 | 157 | 158 | 158 | 158 | 159 | 159 | 159 | 0.0979 | 0.096 | 0.0974 | 0.1015 | 0.1253 | 0.1207 | 0.13 | 0.1347 | 0.0979 |
| 160 | 160 | 160 | 161 | 161 | 161 | 162 | 162 | 162 | 0.1008 | 0.1062 | 0.1005 | 0.1006 | 0.0983 | 0.0917 | 0.1025 | 0.1019 | 0.1008 |
| 163 | 163 | 163 | 164 | 164 | 164 | 165 | 165 | 165 | 0.0774 | 0.0903 | 0.0846 | 0.0827 | 0.0918 | 0.0767 | 0.0794 | 0.0772 | 0.0761 |
| 166 | 166 | 166 | 167 | 167 | 167 | 168 | 168 | 168 | 0.075 | 0.0744 | 0.0787 | 0.0754 | 0.073 | 0.0732 | 0.0754 | 0.0723 | 0.0768 |
| 169 | 169 | 169 | 170 | 170 | 170 | 171 | 171 | 171 | 0.1191 | 0.1183 | 0.1155 | 0.8231^+^ | 0.6686^+^ | 0.2789^+^ | 0.103 | 0.102 | 0.1003 |
| 172 | 172 | 172 | 173 | 173 | 173 | 174 | 174 | 174 | 0.0866 | 0.0758 | 0.0734 | 0.0697 | 0.0707 | 0.0729 | 0.0704 | 0.0695 | 0.0706 |
| 175 | 175 | 175 | 176 | 176 | 176 | 177 | 177 | 177 | 0.0691 | 0.0717 | 0.0699 | 0.0717 | 0.0729 | 0.0693 | 0.0707 | 0.0693 | 0.0726 |
| 178 | 178 | 178 | 179 | 179 | 179 | 180 | 180 | 180 | 0.6162^+^ | 0.541^+^ | 0.5787^+^ | 0.1127 | 0.1096 | 0.1102 | 0.102 | 0.1004 | 0.1127 |
| 181 | 181 | 181 | 182 | 182 | 182 | 183 | 183 | 183 | 0.1105 | 0.1119 | 0.1052 | 0.1021 | 0.1029 | 0.1024 | 0.1136 | 0.1151 | 0.1021 |
| 184 | 184 | 184 | － | － | － | ＋ | ＋ | ＋ | 0.1115 | 0.1126 | 0.1117 | 0.0661 | 0.0711 | 0.0521 | 1.9878^+^ | 2.0598^+^ | 2.1246^+^ |

The left-hand side of the table is seed sample number, and the right-hand side of the table is reading values of ELISA. The reading values right of the table correspond to seed sample number on the left.

“＋” on the lift, positive control; “number” on the lift, seed sample number of positive result; “ number^+^” on the right, positive results.

Each sample was performed for three parallel mechanical repetitions.
