# Supplementary figures and images for: Development of a multiplex RT-PCR assay for simultaneous detection of Cucumber green mottle mosaic virus and Acidovorax citrulli in watermelon
Source: PeerJ. 2019 Aug 22;7:e7539. doi: 10.7717/peerj.7539 (PMC6708580; doi:10.7717/peerj.7539)

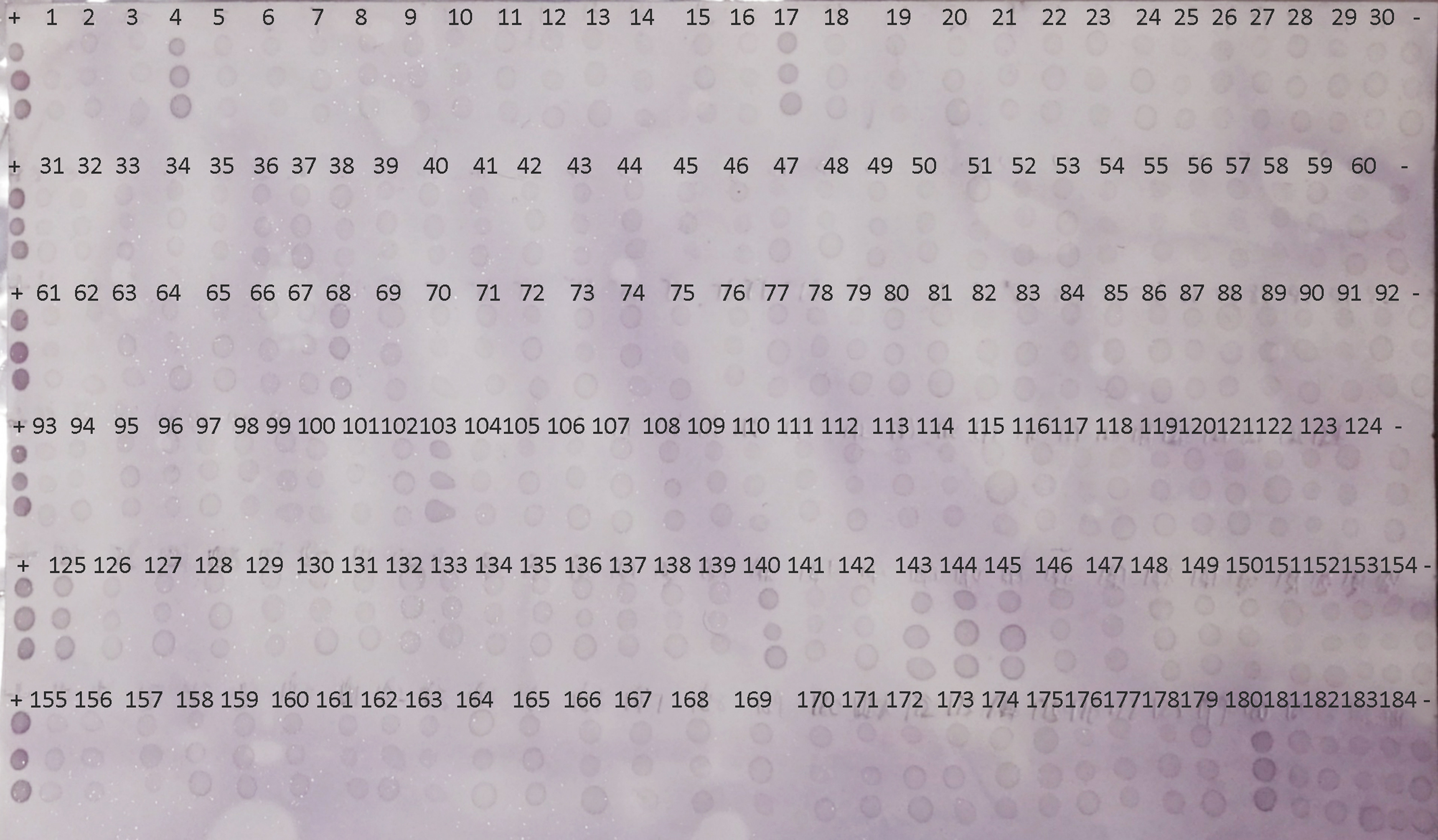

Supplement: Supplemental Information 3 [file peerj-07-7539-s003.png]

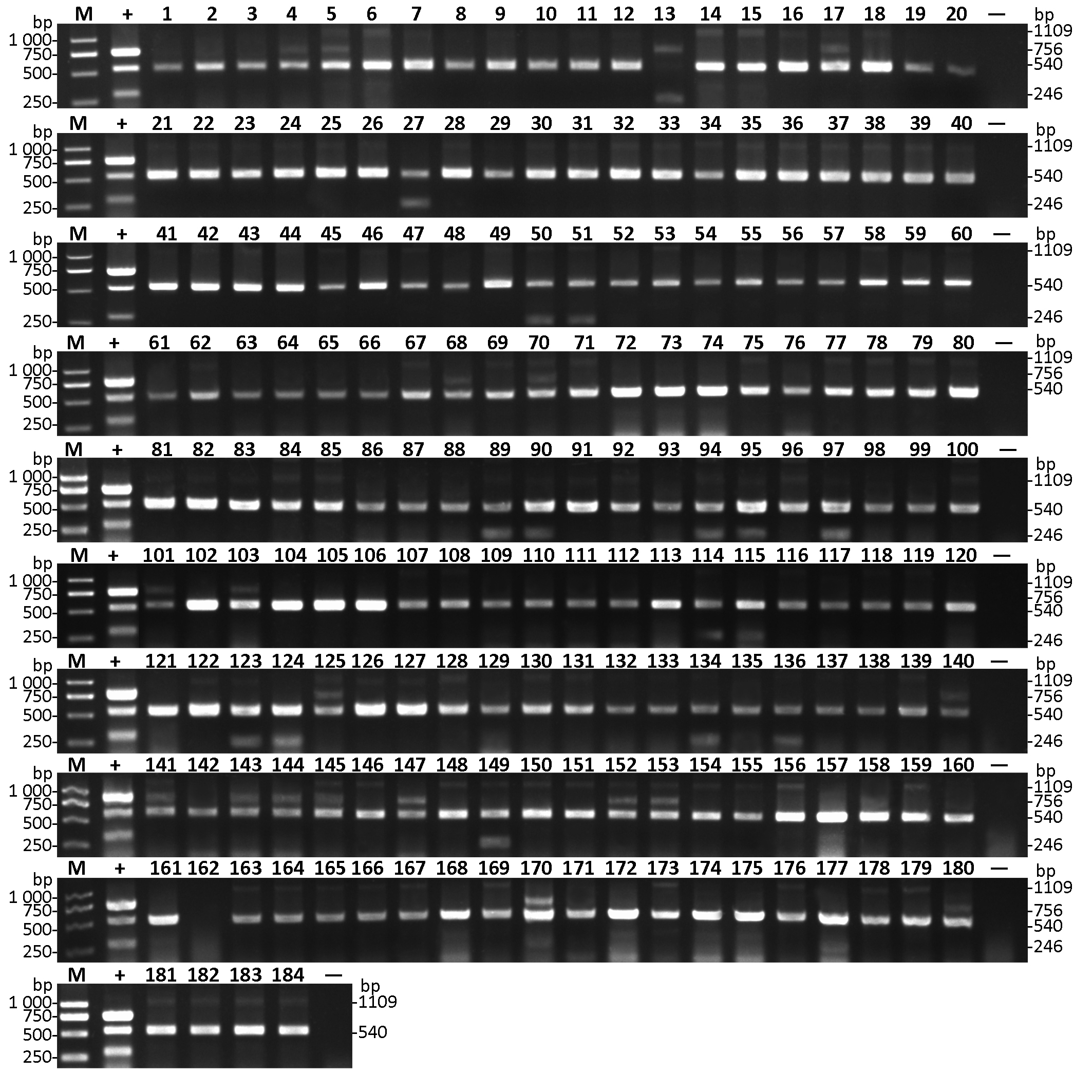

Supplement: Supplemental Information 4 [file peerj-07-7539-s004.png]

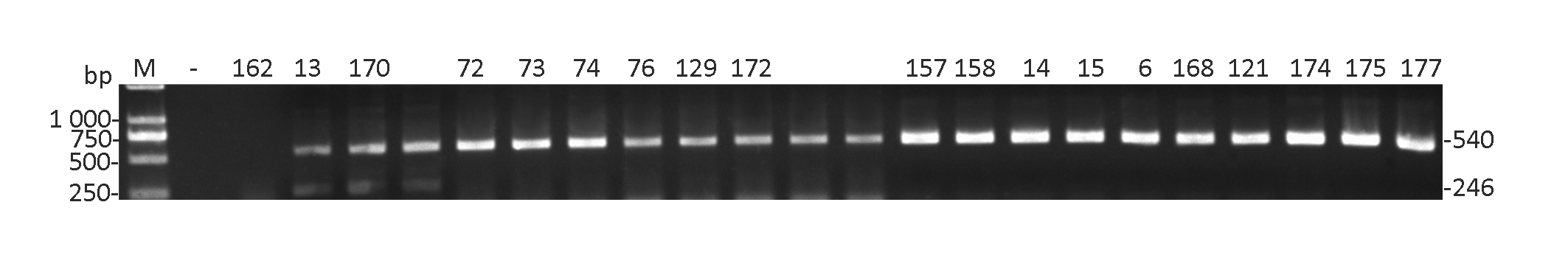

Supplement: Supplemental Information 5 [file peerj-07-7539-s005.png]
